# FMiR: A Curated Resource of Mitochondrial DNA Information for Fish

**DOI:** 10.1371/journal.pone.0136711

**Published:** 2015-08-28

**Authors:** Naresh Sahebrao Nagpure, Iliyas Rashid, Ajey Kumar Pathak, Mahender Singh, Rameshwar Pati, Shri Prakash Singh, Uttam Kumar Sarkar

**Affiliations:** 1 Division of Molecular Biology and Biotechnology, National Bureau of Fish Genetic Resources, Lucknow-226002, India; 2 Division of Fish Taxonomy and Resources, National Bureau of Fish Genetic Resources, Lucknow-226002, India; CSIR Institute of Genomics and Integrative Biology, INDIA

## Abstract

Mitochondrial genome sequences have been widely used for evolutionary and phylogenetic studies. Among vertebrates, fish are an important, diverse group, and their mitogenome sequences are growing rapidly in public repositories. To facilitate mitochondrial genome analysis and to explore the valuable genetic information, we developed the Fish Mitogenome Resource (FMiR) database to provide a workbench for mitogenome annotation, species identification and microsatellite marker mining. The microsatellites are also known as simple sequence repeats (SSRs) and used as molecular markers in studies on population genetics, gene duplication and marker assisted selection. Here, easy-to-use tools have been implemented for mining SSRs and for designing primers to identify species/habitat specific markers. In addition, FMiR can analyze complete or partial mitochondrial genome sequence to identify species and to deduce relational distances among sequences across species. The database presently contains curated mitochondrial genomes from 1302 fish species belonging to 297 families and 47 orders reported from saltwater and freshwater ecosystems. In addition, the database covers information on fish species such as conservation status, ecosystem, family, distribution and occurrence downloaded from the FishBase and IUCN Red List databases. Those fish information have been used to browse mitogenome information for the species belonging to a particular category. The database is scalable in terms of content and inclusion of other analytical modules. The FMiR is running under Linux operating platform on high performance server accessible at URL http://mail.nbfgr.res.in/fmir.

## Introduction

The vertebrate mitochondrial genome (mitogenome) contains 13 protein coding genes, 22 transfer RNA genes, 2 ribosomal (12S small subunit and 16S large subunit) RNA genes and a hyper variable control region (D-loop) in the form of a circular DNA double helix [1]. The mitogenome is widely used as a marker for speciation and evolutionary studies in animals due to its conserved genic region, highly variable control region, high mutation rate and lack of recombination [2,3]. The different mitogenome regions evolve at variable rates and are used for diversity studies at lower taxa levels. The moderately variable cytochrome c oxidase I (COX 1) sequence (~650 bp long) is popularly known as ‘DNA barcode’ and is used for effective species identification [4]. DNA sequences of the less variable ribosomal RNA and moderately variable cytochrome b have been used for phylogenetic studies and for the estimation of evolutionary relationship among species [5–7]. The hyper variable D-loop or control region sequence has been used in population level studies [8,9]. Using the complete mitogenome sequence is a better approach for representing appropriate tree branches in phylogenetic analysis and for resolving taxonomic ambiguities in animals [10,11]. Several features of the mitogenome, such as simple sequence repeat (SSR) function and replication, are still unexplored, and these features can be analyzed by managing mitogenome data in a well-organized database integrated with different analytical modules.

Fish are ecologically and economically important, highly diverse, the largest vertebrate group and offer an almost limitless number of striking examples of evolutionary adaptation to environmental and biotic selection pressure. Fish hold an important position in the evolution of vertebrates on Earth. Globally, more than 32800 species of fishes have been identified [12]. Advances in computational biology and the availability of different fish mitogenome sequences in public repositories provide an opportunity to develop a modern, updated relational database on fish mitogenome resources. Several mitogenome databases are available, including HmtDB [13], mtDB [14] and MITOMAP [15], which focus on a particular taxonomic group for specific analysis. MamMiBase [16] is a database of all mammalian protein coding genes for phylogenetic analysis. MitoFish is a collection of mitogenomes of fish for similarity studies and for the re-annotation of sequences [17]. The mitochondrial genomes of all metazoan species are compiled into Mitome [18] for taxonomic position and homology pattern searching. MitoZoa is another well-organized database for comparative and evolutionary analysis [19]. The NCBI Organelle Genome Resource [20] and GOBASE [21] databases contain organelle genomes from all taxonomic groups.

The Fish Mitogenome Resource (FMiR) is a new database of curated fish mitogenome sequences developed for exploring valuable genetic information and facilitating sequence analysis. The database has been developed using LAMPP (Linux-Apache-MySQL-PHP-Perl) technology and NCBI [22] and FishBase [12] data resources. The present contribution describes the development of FMiR and its functional capabilities. The relational database currently contains mitogenome sequences of 1302 globally distributed species belonging to 297 families and 47 orders. The database also covers other species information, such as taxonomy, conservation status, habitat, distribution and occurrence. FMiR provides the workbench for fetching records, finding SSRs in the mitogenome and designing primers for microsatellite loci. The similarity search tools have also been included in the FMiR for sequence annotation and for the comparative study of genes, proteins and other sequences. FMiR could be useful to researchers by assisting with interspecific and intraspecific characterization, population genetics and the identification of species-specific or habitat-specific SSRs.

## Material and Methods

### Data source and parsing

**Fig 1:**
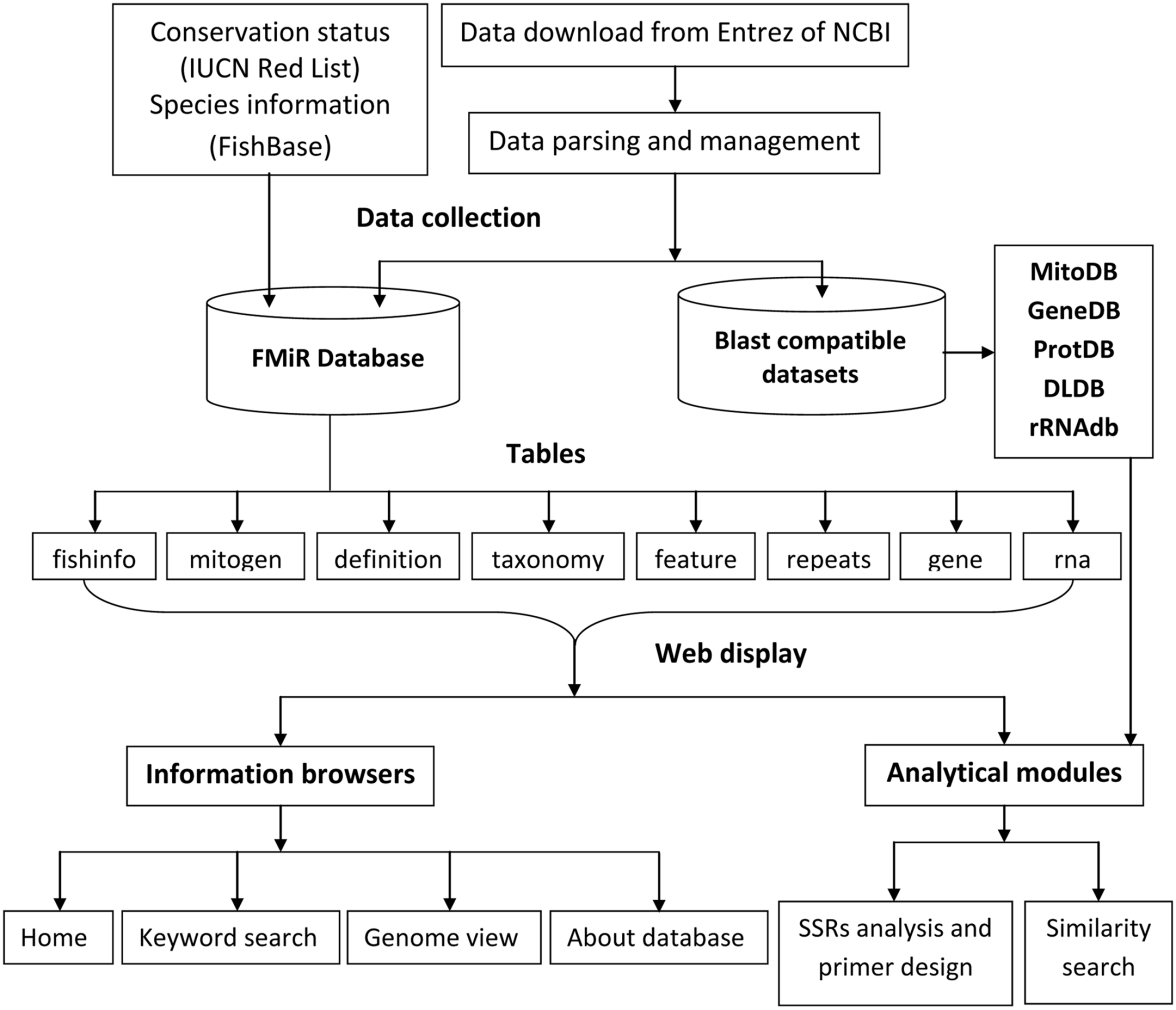
Architecture and dataflow diagram of FMiR.

Fish mitogenome sequences were downloaded from NCBI using the Entrez [23] query ‘Fish mitochondrion complete genome’ under nucleotide search option. The presently available GenBank and FASTA format files of the fish mitogenomes were downloaded for annotation and sequence records, respectively. Priorities were given to mitogenome sequences having RefSeq entries [24]. Thirty fish species sequences belonging to the Indian sub-continent unavailable in RefSeq were taken from GenBank [25]. The other species information, such as habitat, distribution and common name, were downloaded from FishBase, and conservation status was obtained from IUCN Red List [26]. A Perl parsing program (InformationParser.pl) was written and applied to extract information from the downloaded files according to database schema and to manage data into database. The data flow and complete architecture of FMiR has been presented in Fig 1. This program supports management and content updates of FMiR based on the availability of new data arrivals in RefSeq/GenBank.

### Hypothetical annotation

The annotation information regarding the D-loop region of a species not defined in the ‘.gb’ file was annotated by applying stand-alone Blast [27]. During this process, a Blast compatible database ‘dloopdb’ was created of defined D-loop sequences through the ‘formatdb’ program of the Blast package. Thereafter, ‘AlignmentAnalysis’ [28] was applied on ‘dloopdb’ for obtaining homologous sequences at a high threshold value. This program aligns query sequences with ‘dloopdb’ using the Blastn program and extracts homologous sequences from the alignments. The sequences of 167 species that had no information on the D-loop region were used as query sequences for ‘dloopdb’ by the program. The region of the query that aligned with the D-loop-targeted sequences on a high similarity threshold (>80% identity) was assumed as the D-loop region for that query. Hypothetical D-loop regions were predicted for sequences that had no previous annotation information. Annotated D-loop sequences were appended into the main database as well as ‘DLDB’ (Blast compatible sequences dataset). The hypothetically annotated D-loop sequences can be browsed through the 'About database' menu item that displays annotated D-loop sequences in a table.

### Design and development

#### Database

**Fig 2:**
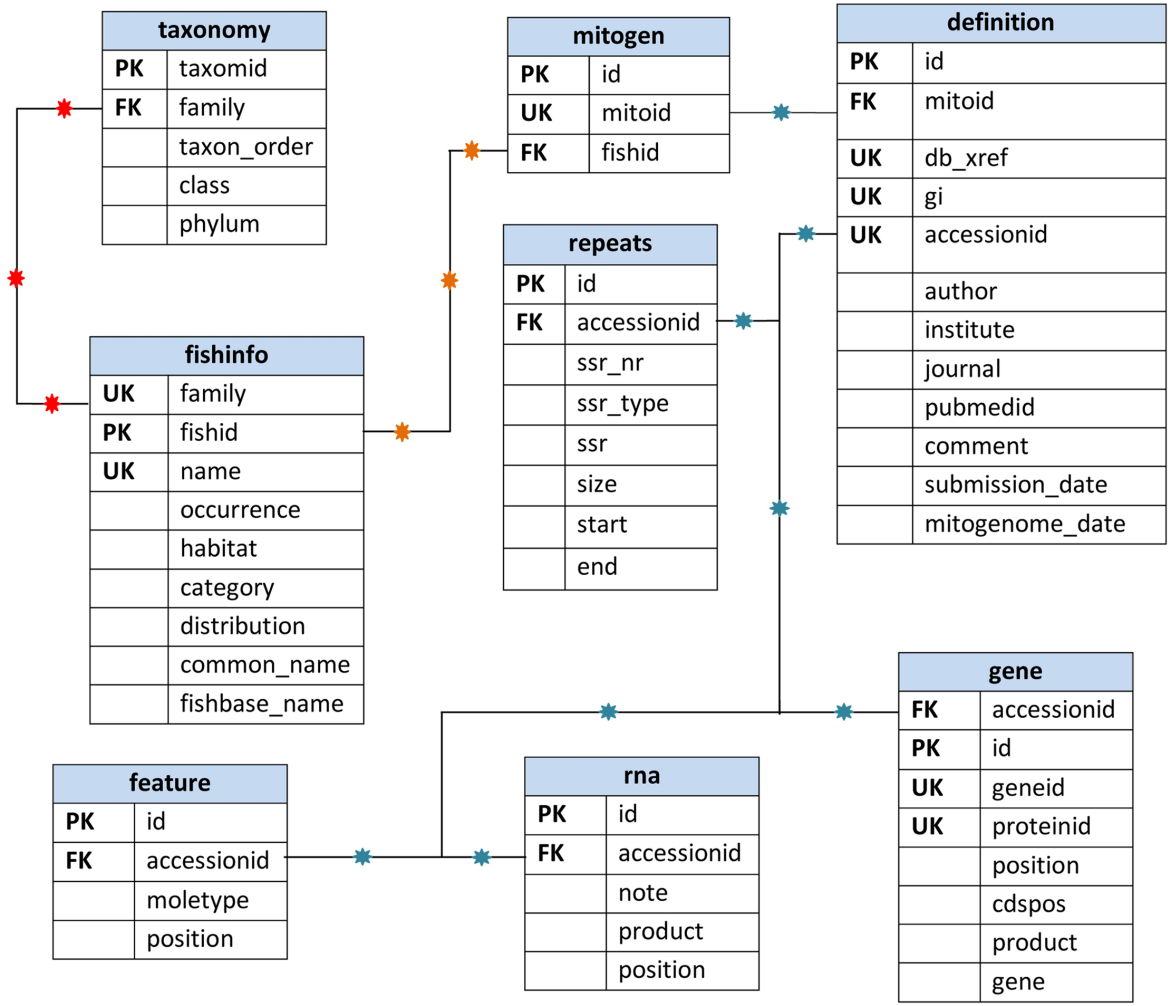
Entity relationship diagram of the FMiR database.

MySQL, a relational database management system, was used to design and develop the database under Linux operating platform on Intel Xeon based high performance computer server. Tables were designed and relationships among the tables were created using unique, primary and foreign keys. Fig 2 shows the entity relationship (E-R) model of the database along with attributes covered in each table.

The table ‘fishinfo’ covers information on taxon, biogeography and conservation status. The table ‘definition’ holds detailed molecular information on the whole mitogenome. The table ‘mitogen’ works as a bridge between the tables ‘fishinfo’ and ‘definition’. The table ‘taxonomy’ covers the systematic species information. Tables ‘gene’, ‘rna’ and ‘feature’ cover information on gene, RNA and D-loop sequences, respectively. Finally, the table ‘repeats’ covers all of the microsatellites repeats generated by ‘MISA’ [29] (Fig 2).

#### Web interface

**Fig 3:**
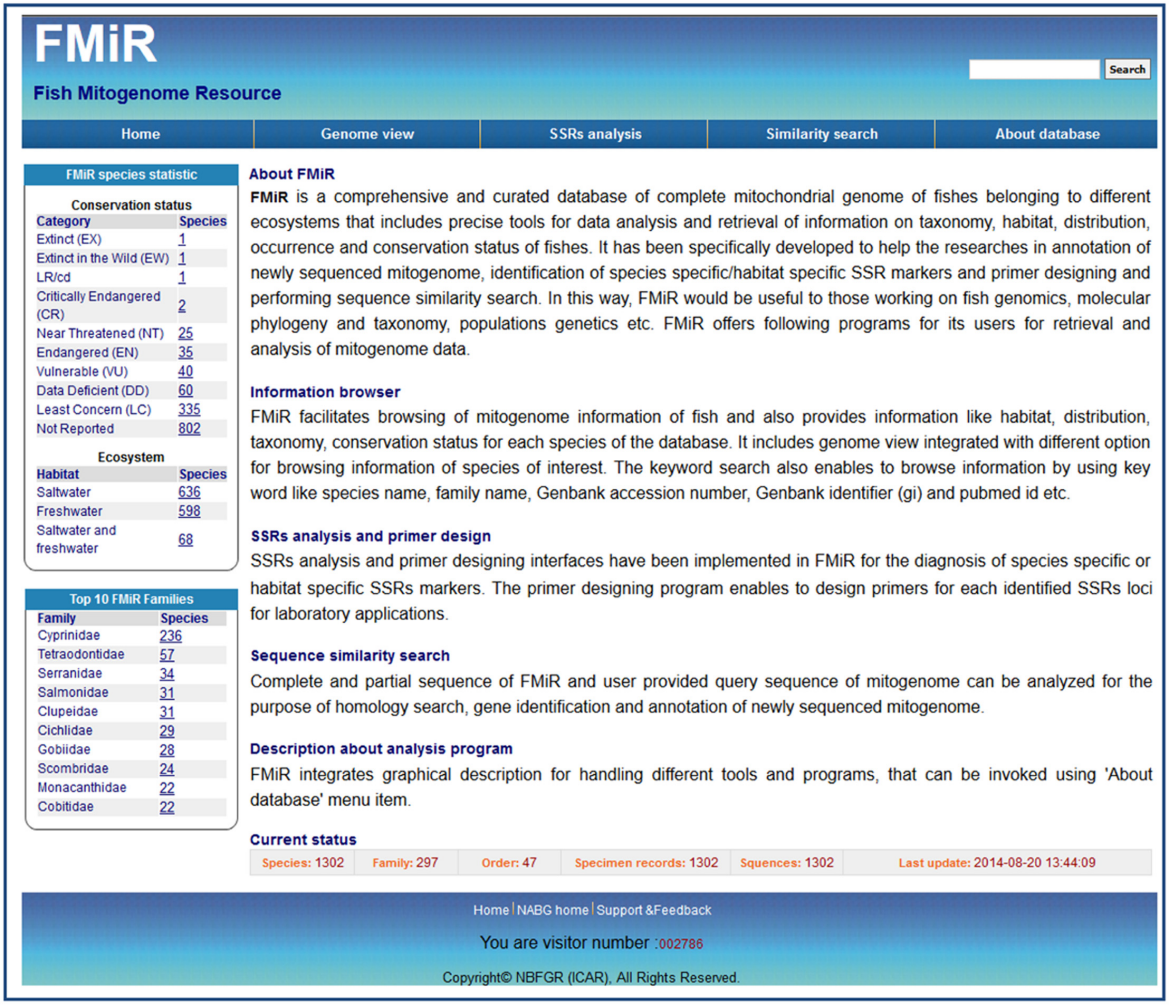
FMiR home page.

For the web-based delivery of information and analysis, user interactive web interfaces were designed and implemented using PHP (Pre hypertext processor), Perl, DBI (database interface), CGI (Common Gateway Interface), JavaScripts, CSS (cascading style sheets) and HTML technologies. The different tools integrated with the database were also developed and implemented in the web interface to provide a workbench for searching, browsing and analyzing mitogenome sequences from the database (Fig 3). The different analytical tools for mitogenome analysis are sequence alignments, locating SSRs and designing primers.

### Design and implementation of analytical tools

#### Similarity search

Blast algorithms were implemented for querying either a user-provided sequence or a sequence already stored in the FMiR database for similarity analysis against complete mitogenome sequences, subsequences (protein coding genes, rRNA genes and D-loop) and protein sequences. A program ‘SubParse’ was developed in Perl and applied for parsing genes (protein coding, ribosomal RNA), D-loop and protein sequences in FASTA format from the downloaded files ‘.gb’ for annotation and ‘.fasta’ for sequences. These parsed sequences were used for developing Blast-compatible target datasets by using the program ‘formatdb’ of the Blast suite to perform alignment between query and target sequences (Fig 1). The Blast-compatible datasets were programmed for automatic updating and synchronizing. These Blast-compatible datasets use different Blast programs for similarity searches, such as Blastn (nucleotide query against nucleotide dataset), Blastp (protein query against protein target) and Blastx (nucleotide query against protein datasets) (Table 1). The similarity search can be performed by either of the two methods. In the first method, the provision has been made for selecting the dataset (gene, protein and other nucleotides) that automatically activates the corresponding Blast program for the selection of sequence type, such as gene, protein, rRNA and D-loop. The query species can be selected from the drop down menu. This module is restricted to Blast the sequences from FMiR only. The second method uses the user-provided query sequence against the particular dataset by setting the parameters accordingly.

**Table 1:** Various Blast-compatible datasets.

Dataset name	Sequences	No. of sequences	Program Compatibility
MitoDB	mitogenome	1302	Blastn
GeneDB	Genes	16917	Blastn
ProtDB	Proteins	16917	Blastx, Blastp
DLDB	D-loop	1279	Blastn
rRNAdb	rRNA	2604	Blastn

#### Sequence diversity analysis

The inputs to the implemented Blastn program are word size, percent identity and maximum number of alignments; the default setting of these inputs are 25 bp, 85% and 250, respectively. The higher identity percent (>85%) and longer word size (>25 bp) can be used to deduce sequence diversity among congener or intra family species, whereas smaller word size and low identity percent deduce sequence diversity among interfamily species and higher taxon level species. This module also provides the ability to deduce particular subsequence (gene, D-loop and rRNA) conservation across species.

#### SSRs analysis and primer designing

'MISA' was used to investigate simple and compound SSRs in complete mitogenome and isolated subsequences files (gene, rRNA and D-loop) for determining occurrence, frequency and repeat types. SSRs with a minimum length of twelve nucleotides have been widely used in repeat finding studies [30–32]. During SSR finding, the input parameters for 'MISA' were set to twelve contiguous repeats for mononucleotides motifs, six repeating units for dinucleotide motifs, four repeating units for trinucleotide motifs and three contiguous repeats for tetra-, penta-, and hexanucleotide motifs. The program generates two output files of extensions ‘.misa’ and ‘.statistics’ that cover SSRs and SSR statistics, respectively.

Primer3, a primer designing program [33,34], computed multiple sets of forward and reverse primers for microsatellite loci along with information on melting temperature (Tm), GC content, start and end position and product size. These primers can be used in PCR for the identification of loci and for genotyping individuals.

## Results and Discussion

FMiR covers curated mitogenomes of 1302 endemic, commercial and aquaculture-important fish species belonging to 297 families and 47 orders. This web-based framework integrates different tools that provide a workbench for browsing, retrieving and analyzing mitogenome data. The ‘Home page’ section covers different tools and information on the database. In addition, the ‘Home page’ provides information on the number of species included in the top ten families, conservation status and ecosystem-based listing of species. The tools for analyzing SSRs, primer design, sequence similarity and keyword searches enhance the utility and importance of the database (Fig 3). The menu item 'About database' presents graphical views and textual help for different tools.

### Browsing information

Through the 'Genome view' menu item of the web interface, information on species of interest can be browsed country wise using other selection criteria, such as habitat, conservation status and occurrence.

‘Genome view’ presents a tabular view covering species name and mitogenome information including base composition percentage, number of genes, transfer RNA, ribosomal RNA, NCBI accession number and PubMed ID. A Blast link corresponding to each species record in the table provides a way to perform alignments based on complete or selected fragments of a query sequence. Moreover, each species name has a link that provides information, such as the family, common name, synonyms, habitat, occurrence, conservation status, distribution and taxonomy of the species in a popup window. The NCBI accession number and PubMed ID for each specimen record have also links. The ‘Top 10 FMiR families’ and ‘FMiR species statistics’ in the home page provide other means of viewing similar species information.

### Keyword Search

The keyword search accepts keywords such as species name, common name, family name, accession number, GenBank identifier and PubMed ID for retrieving information from the database. This search result displays different views based on the keyword to present the information.

### Repeat analysis and primer designing

SSRs analysis and primer designing were implemented in FMiR using methodologies described in FishMicrosat [30]. The 'SSRs Analysis' menu item provides the ability to view SSRs in the mitogenome. The analysis result provides information on species name, family name, NCBI accession, SSR number, location in the mitogenome, SSRs and their position in mitogenome and a link for primer design. In this page, a dynamic drop down box listing repeat motifs of all available SSRs in the database was implemented to view the information using mono- to hexa-nucleotide repeats options. ‘SSRs analysis’ provides statistics of different repeat types and their distribution in particular regions such as genes, D-loop and rRNAs. The ‘Primer’ button leads to the ‘Primer design’ page that facilitates primer design and viewing repeat information. The implemented ‘Primer3’ program designs primers for sequences having SSRs and ensures a suitable flanking region length and sufficient GC content. The ‘Primer design’ page provides information on the query specimen, SSRs containing the mitogenome subsequence of multiple primers along with Tm values, GC content, start to end position and PCR product size. These designed primer sequences would be useful for analyzing loci across species through PCR amplification. The SSR analysis and primer design tools for repeat loci were unavailable in earlier animal mitogenome databases, and FMiR is the first web resource for facilitating SSR mining in mitogenome and designing primers for the repeat locus. The repeat loci can be used as species-specific markers after PCR validation [35,36]. The COI gene in two closely related species contained in the database, *Puntius tetrazona* and *Puntius ticto*, contains significantly different SSRs, (CTT)4 and (TAC)4, respectively, that would be helpful for developing *P*. *tetrazona*- or *P*. *ticto*-specific PCR markers. SSRs can also be used as marker if SSRs exist identically across broad taxonomic groups [36,37]. In concordance with these studies, (AACC)3 repeats were observed in the ATP6 gene in several marine species belonging to different families and (TA)n polymorphic repeats were mainly observed in freshwater species, especially in *Cyprinidae*. Thus, targeted primers for the (AACC)3 or (AT)n loci in PCR amplifications could be used to identify marine and freshwater ecosystem species.

### Repeats estimation

**Fig 4:**
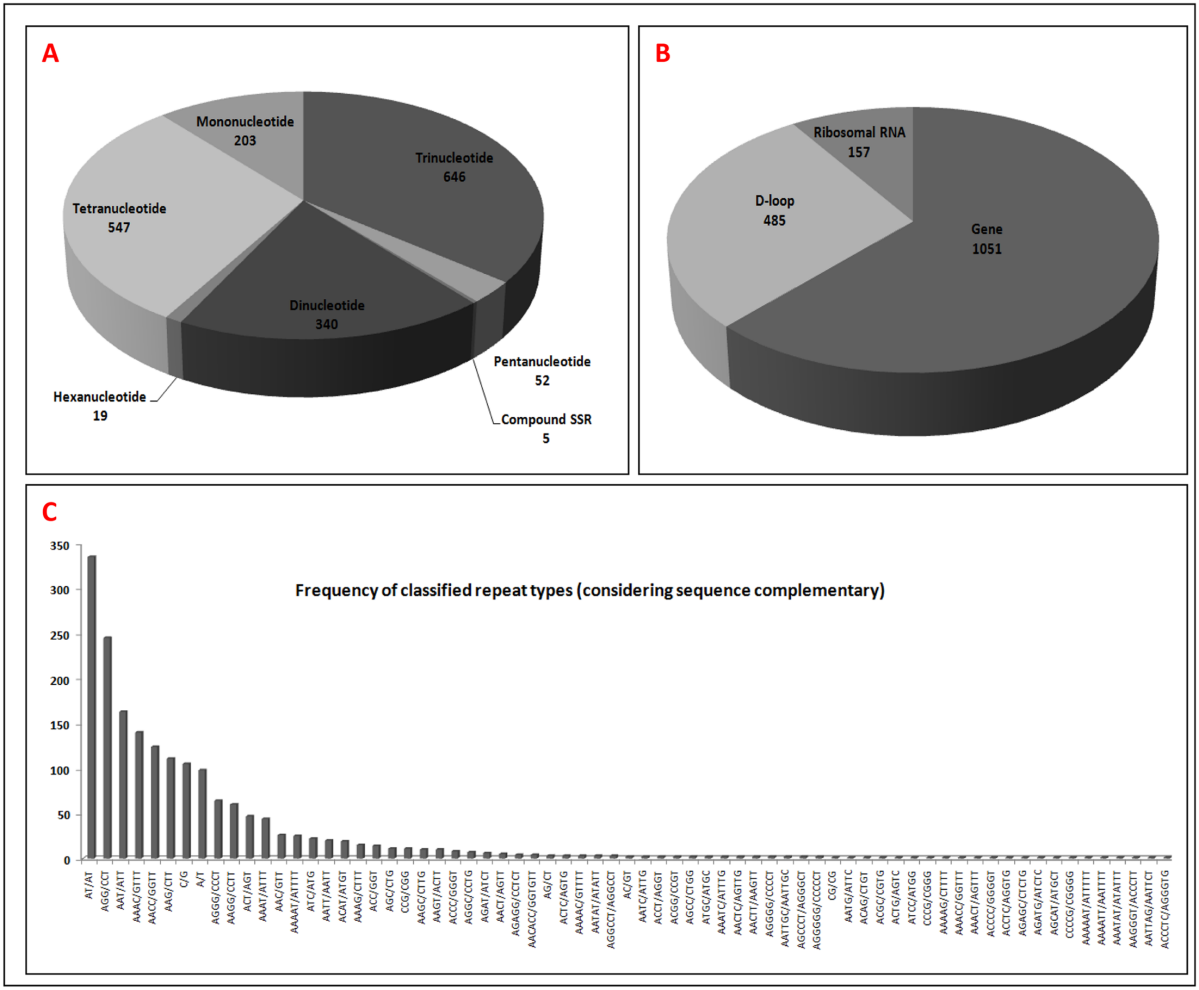
SSR distribution in the mitogenome sequences of FMiR. (A) Sum of SSR frequencies in individual SSR types (mono- to hexa- and compound) (B) Total SSRs observed in subsequences (C) Frequencies of all observed SSRs with their complementary motifs.

The 'SSRs Analysis' menu item also provides information on the total numbers of repeats, repeats in particular subsequences and frequently occurring repeats. Presently, the FMiR database covers 1812 SSRs in different combinations of mono- to hexa nucleotide repetitive motifs. Among these SSRs, frequencies of tri- (646) and tetra- (547) nucleotide repeats were comparatively higher and were detected mostly in the protein coding genes and in ribosomal RNA. The repeat frequency (340) of dinucleotides was found to be highest in the D-loop region. Other repeat types, mono-(203), penta-(52), hexa-(19) and compound SSRs (5), had fewer occurrences (Fig 4A). A total of 1051 repeats were found in the protein coding genes, 485 SSRs were found in the D-loop regions, and 157 repeats were found in the ribosomal RNA of the mitogenome (Fig 4B). The occurrences of the most frequent and rare nucleotide repeats were calculated. Di-nucleotide repeats AT/TA with 335 occurrences and tri-nucleotide repeats AGG|CCT with 245 occurrences were the most common among all di- and tri-nucleotides repeats, while tetranucleotide repeats AAAC/GTTT, with 140 occurrences, were the most common among this class of repeats. (Fig 4C).

### Similarity search

This search program provides multiple options for searching sequence similarity against the FMiR datasets (Table 1). The alignment analysis can be carried out using internal and external query sequences separately against complete or specified mitogenome regions.

### Internal queries

Any sequence from FMiR (mitogenome, subsequence and protein sequence) can be used as a query sequence for performing alignment analysis with different datasets. The homology search for the complete mitogenome of internal sequences can be processed through the ‘Genome view’ menu that provides a link for the Blast of each species record. The Blast of a query species provides an alignment summary with respect to the selected query. The species displayed on the alignment summary page are the targeted species for the selected query species. A link to the targeted species provides a sequence alignment view with the query species in a popup window. The alignment summary page also displays input parameters for reselecting the query species with a specified mitogenome region. Sequence alignment for subsequences can be performed in the same way, and an alignment summary can be viewed.

The sequence similarity search for internal subsequences can be found through the menu item ‘Similarity search’. The program accepts three input parameters: (1) selection of dataset (gene, protein and other sequences), (2) selection of query sequence type (gene from the gene list, protein from the protein list and D-loop, 16S and 12S rRNA from the other nucleotide list) and (3) selection of query species. The other user input parameters, such as maximum alignment number, identity percent cut off and word size, can also be used for a similarity search against nucleotide datasets, whereas the protein dataset uses default parameters. The specified subsequence or protein sequences of selected species can be queried against the appropriate dataset, as listed in Table 1. The query result provides an alignment summary and a link to the species name that leads to an alignment view.

#### External queries

To query external sequences with the different FMiR datasets, the homology search method was implemented and can be accessed through the link 'External query sequence analysis' provided in the left portion of the ‘Similarity search’ page. This query invokes a window for pasting the query sequence in FASTA format along with input parameters. Four input types are required for performing the alignment: (1) the selection of a target dataset as listed in Table 1, (2) the selection of a dataset-compatible Blast algorithm, (3) the selection of a mitochondrial sequence (DNA or protein) and (4) an input sequence in FASTA format. User input parameters such as maximum alignment number, identity percent cut off and word size can also been provided using Blastn, whereas Blastp and Blastx programs use default parameters. The submission of these inputs through the submit button performs an alignment with FMiR sequences. If parameters are not selected appropriately, the program terminates by displaying a warning message. FMiR is useful for the analysis of mitochondrial sequences segments and translated products on a single platform and annotates and re-annotates mitogenome genes and D-loop regions.

The similarity search also facilitates alignment of partial mitogenome of mammals such as coding genes, rRNAs and D-loop region using appropriate selection parameters like lower identity percentage and small word size. In this way, it identifies the conserved regions of mitogenome across lower to higher taxon and can be useful for studying evolution of genes across animal group [38,39]. The complete alignment of mammalian and fish mitogenome may not be feasible due to distant relationship and variation in gene organization.

The annotation of the query sequence can be achieved in subsequent alignments using a particular dataset, such as gene, rRNA and D-loop, and the result represents the location of particular genes, rRNA and D-loop regions, which is helpful for the comparative analysis of partial and complete genomes and for determining genetic divergence across species.

The similarity search for internal and external queries for complete as well as mitogenome subsequences were included in FMiR by considering their significance in phylogenetic studies. Among notable studies, Gerber (2001) reviewed the different mitogenome regions and their significance for estimating various phylogenetic relationships at different taxon levels (population to phyla). 12S ribosomal RNA genes are highly conserved, and they are used at the phyla to subphyla levels for evolutionary estimation. 16S ribosomal DNA has been used in phylogenetic study at mid taxon levels such as family and genera. Protein coding genes COI, Cyto b and D-loop sequences are powerful markers for deducing evolutionary relationship at the genera, species and population levels [40]. The phylogenetic studies based on complete mitochondrial genome in fish have resolved the evolutionary ambiguities but lack the actual information on frequent mutable regions in particular subsequences across different taxonomic groups [41,42]. The similarity search in FMiR provides the ability to analyze different parts of the mitogenome at different taxon levels. It has been found that 12S rRNA is more conserved than the D-loop, and this result supports work conducted by earlier researchers. Carapelli (2007) used sets of protein coding genes along with protein sequences for confirming the reciprocal paraphyly of Hexapoda [43]. Similarly, Miya (2003, 2000) used the protein coding genes and complete mitogenomes in two separate studies for the phylogenetic examination in fish [41,11]. FMiR is a flexible platform for analysis of protein sequences and individual component of mitogenome, thus enhancing its utility in phylogenetics. Further FMiR enables to deduce conservation of particular subsequence across the species.

## Conclusion

FMiR is a new database of curated fish mitochondrial sequences and presently covers mitogenome information of 1302 globally distributed fish species belonging to 297 families and 47 orders reported from saltwater and freshwater ecosystems. The database also contains other valuable information on each fish species such as habitat, distribution, occurrence, conservation status and taxonomy. FMiR is the workbench for finding SSR motifs, repeat orientation, sequence similarity search and annotation, identification of repeats loci and primer design. In addition, the database presents the relative abundance of microsatellite repeats in the mitogenomes. It is also possible to compare complete and individual components of genomes for deducing variation across fish species. Thus, the FMiR workbench has bidirectional facilities for species identification using either the mitogenome sequence or using species-specific markers for experimental validation, which can play an imperative role in cutting edge areas of research, such as marker selection, speciation, evolutionary studies, genetic relatedness among the species and genetic improvement programs of commercially important aquaculture species. The database supported by the dedicated researchers of the Institute is getting updated with the new release of fish mitogenome information in NCBI. The NBFGR has developed the capacity and required infrastructure to manage and maintain fish genomic databases.
